# Evaluation of Ovarian Reserve Tests and Age in the Prediction of Poor Ovarian Response to Controlled Ovarian Stimulation—A Real-World Data Analysis of 89,002 Patients

**DOI:** 10.3389/fendo.2021.702061

**Published:** 2021-08-30

**Authors:** Xue Wang, Lei Jin, Yun-dong Mao, Juan-zi Shi, Rui Huang, Yue-ning Jiang, Cui-lian Zhang, Xiao-yan Liang

**Affiliations:** ^1^Reproductive Medicine Center, Henan Provincial People’s Hospital, People’s Hospital of Zhengzhou University, Zhengzhou, China; ^2^Reproductive Medicine Center, Tongji Hospital of Tongji Medical College of Huazhong University of Science and Technology, Wuhan, China; ^3^State Key Laboratory of Reproductive Medicine, Clinical Center for Reproductive Medicine, The First Affiliated Hospital of Nanjing Medical University, Jiangsu Province Hospital, Nanjing, China; ^4^Reproductive Medicine Center, Northwest Women’s and Children’s Hospital, Xi’an, China; ^5^Reproductive Medicine Center, Sixth Affiliated Hospital of Sun Yat-sen University, Guangzhou, China

**Keywords:** poor ovary response, *in vitro* fertilization/intracytoplasmic sperm injection, female age, real-world study, ovarian reserve tests

## Abstract

**Aims:**

This study aimed to explore the value of ovarian reserve tests (ORTs) for predicting poor ovary response (POR) and whether an age cutoff could improve this forecasting, so as to facilitate clinical decision-making for women undergoing *in vitro* fertilization (IVF).

**Methods:**

A retrospective cohort study was conducted on poor ovary response (POR) patients using real-world data from five reproductive centers of university-affiliated hospitals or large academic hospitals in China. A total of 89,002 women with infertility undergoing their first traditional ovarian stimulation cycle for *in vitro* fertilization from January 2013 to December 2019 were included. The receiver operating characteristic (ROC) curve was performed to estimate the prediction value of POR by the following ORTs: anti-Mullerian hormone (AMH), antral follicle count (AFC), basal FSH (bFSH), as well as patient age.

**Results:**

In this retrospective cohort, the frequency of POR in the first IVF cycle was 14.8%. Age, AFC, AMH, and bFSH were used as predicting factors for POR, of which AMH and AFC were the best indicators when using a single factor for prediction (AUC 0.862 and 0.842, respectively). The predictive values of the multivariate model included age and AMH (AUC 0.865), age and AFC (AUC 0.850), age and all three ORTs (AUC 0.873). Compared with using a single factor alone, the combinations of ORTs and female age can increase the predictive value of POR. Adding age to single AMH model improved the prediction accuracy compared with AMH alone (AUC 0.865 *vs.* 0.862), but the improvement was not significant. The AFC with age model significantly improved the prediction accuracy of the single AFC model (AUC 0.846 *vs.* 0.837). To reach 90% specificity for POR prediction, the cutoff point for age was 38 years old with a sensitivity of 40.7%, 5 for AFC with a sensitivity of 55.9%, and 1.18 ng/ml for AMH with a sensitivity of 63.3%.

**Conclusion:**

AFC and AMH demonstrated a high accuracy when using ROC regression to predict POR. When testing is reliable, AMH can be used alone to forecast POR. When AFC is used as a prediction parameter, age is suggested to be considered as well. Based on the results of the cutoff threshold analysis, AFC ≤ 5 and AMH ≤ 1.18 ng/ml should be recommended to predict POR more accurately in IVF/ICSI patients.

## Introduction

Predicting a patient’s ovarian response prior to the start of the first IVF cycle is important in clinical practice for providing important diagnostic and prognostic value.

Poor ovary response (POR) is characterized by a low number of growing follicles and low serum estradiol levels after exogenous gonadotropin stimulation, resulting in a poor oocyte retrieval. POR is associated with poor reproductive outcomes (1, 2). According to the consensus elaborated by the European Society of Human Reproduction and Embryology (ESHRE) in 2011, to define POR, at least two of the following three features must be present: (i) Advanced maternal age (≥40 years) or any other risk factor for POR, (ii) a previous POR (≤3 oocytes with a conventional stimulation protocol), (iii) an abnormal ovarian reserve test (i.e., AFC of 5–7 follicles or AMH of 0.5–1.1 ng/ml). Two episodes of POR after maximal stimulation are sufficient to define a patient as a poor responder in the absence of advanced maternal age or abnormal ORT. From that time, according to the literatures, the prevalence of POR after ovarian stimulation ranged from 5.6 to 35.1% worldwide (3), and it relates to poor IVF outcomes and low pregnancy rate for these patients (4).

In the past few decades, numerous studies have been carried out to measure ovarian reserve through ovarian reserve tests (ORTs) (5–10). Basal FSH (bFSH) plus estradiol levels or AMH are recommended as most appropriate ovarian reserve screening tests. According to increasing numbers of studies, AMH and antral follicle count (AFC) represent direct and accurate measurements of the ovarian follicle pool (11, 12). ORTs are often used in combination to improve the prediction of POR. However, according to the past meta-analysis (6, 13), combinations of a few tests only show a minimal improvement in prediction of POR when compared with using a single test. The lack of improvement might be explained by the heterogeneity of the tests and the cutoff points used in different research studies. Furthermore, ORTs only define ovarian reservation quantitatively, while the best surrogate marker for oocyte quality is age (6, 14, 15). ORT results, combined with age, could be useful for discussing a patient’s prognosis and recommending a treatment plan in practice. Thus, in order to forecast POR, using age in addition to ORTs should be investigated. More specifically, an optimal combination of measurements should be determined, which considers differences in methods of measuring hormone and the definition of uniform POR.

The study benefited from the establishment of a multicenter retrospective database and used a large sample of 89,002 patients who underwent their first *in vitro* fertilization (IVF) cycles in China to analyze the accuracy of POR prediction by female age and ORTs alone and in combination. The study also explored the cutoff points of key indicators to predict POR and stratified cutoff point according to age.

## Methods

### Study Cohort and Data Acquisition

This study included the first oocyte retrieval IVF/ICSI cycle of all patients from January 2013 to December 2019 at five reproductive centers in university-affiliated hospitals or large academic hospitals in China including the Sixth Affiliated Hospital of Sun Yat-sen University, Henan Provincial People’s Hospital, Jiangsu Provincial People’s Hospital, Tongji Hospital of Tongji Medical College of Huazhong University of Science and Technology, and Northwest Women’s and Children’s Hospital. The study was reviewed and approved separately by the ethical committees in each hospital, namely, the Reproductive Medicine Ethics Committee of Henan Provincial People’s Hospital (SYSZ-LL-2019110401), Medical Ethics Committee of Tongji Hospital of Tongji Medical College of Huazhong University of Science and Technology (TJ-IRB20210320), Medicine Ethics Committee of Jiangsu Provincial People’s Hospital (2020-SR-046), Medical Ethics Committee of the Sixth Affiliated Hospital of Sun Yat-sen University (2020ZSLYEC-295), and Medical Ethics Committee of Northwest Women’s and Children’s Hospital (2019013). The need for individual consent was waived by the committees due to the retrospective character of the study. Data was desensitized to hide personal information before being processed.

The raw data came from the IVF database of the five reproductive centers. We retrieved the desensitization data of patients who underwent IVF/ICSI treatment from January 2013 to December 2019 from each center. The types of raw data collected included hospital admission summary sheet, medical history records of the couples, cycle information, ovulation monitoring, oocyte retrieval records, embryo culture records, frozen and thawing records, transplant records, follow-up records. Data were processed from medical records into standardized research datasets for further analysis.

### Inclusion and Exclusion Criteria

Inclusion criteria: Female patients with regular menstruation and bilateral ovaries at one of the five reproductive centers with first-time fresh cycles of IVF from January 2013 to December 2019 were included in the analysis.

Exclusion criteria: Patients with evidence of any of the following conditions were excluded from the study: ① polycystic ovarian syndrome (PCOS) (according to Rotterdam Criteria); ② history of ovarian surgery; ③ history of chemotherapy and pelvic radiotherapy; ④ pretreatment of oral contraceptives within 2 months before conducting the IVF cycle; ⑤ natural cycle IVF and mild stimulation cycle with daily gonadotropin (Gn) <150; ⑥ canceled oocyte retrieval cycle that isn’t due to poor ovarian response.

### Treatment

Every patient that met the inclusion criteria underwent the first *in vitro* fertilization cycle. The stimulation protocol and the dose of gonadotropin were determined by the reproductive endocrinologist. In all cases, the dose of gonadotropin was chosen to optimize the number of oocytes retrieved while minimizing the risk of ovarian hyperstimulation syndrome (OHSS).

Before the cycle, venous blood was collected on days 2–4 of the menstrual cycle, and the AFC was measured through a transvaginal ultrasound examination by a reproductive endocrinologist or an experienced sonographer. Within one center, these posts are filled by relatively permanent personnel. Since all the five reproductive centers are large artificial reproductive technology centers of China and each center has its own personnel training and assessment process, thus, the results of the AFC were reliable. AFC is defined as the number of 2–10 mm diameter follicles in two ovaries. After standard venipuncture, the blood sample was completely coagulated and the sample was centrifuged. Then 1 ml serum was removed to a new tube, frozen at 2–8°C within 24 h after blood collection, and tested in an independent laboratory of each IVF center within 2 days. Kangrun Biotech Reagent Automatic SMART6500 immunoassay analyzer was used to detect levels of AMH and sex hormones in serum and plasma samples. The published total imprecision of the AMH assay kit was 2.4–5.2% (16, 17).

### Definition and Statistics

POR is defined as the cancelation of the oocyte retrieval cycle due to poor ovarian response or cycles in which the number of oocytes retrieved is three or fewer (5, 6, 18). ORTs include bFSH, AMH, and AFC.

POR was designated as the dependent variable (1=POR; 0=enough to achieve high ovarian response), and the following continuous variables were used as independent variables in the analysis: AMH, AFC, bFSH, age. Receiver operating characteristic (ROC) analysis was used to map the sensitivity and specificity of the four independent variables, in order to predict the POR of all possible cutoff points for each indicator. The area under the ROC curve and 95% CI were then described. According to previous studies (3, 18), the cutoff points are typically determined to be the value when the specificity of predicting POR is 90%. Maximizing specificity was the goal in this study in identifying a cutoff point for predicting POR to avoid overestimating the risk of POR (6, 11). The ideal screening test should demonstrate high specificity to minimize the risk of a false-positive determination of decreased ovarian reserve in a woman with normal ovarian reserve (11, 18). Therefore, the cutoff point that maximizes specificity is preferred, even if it means reduced sensitivity. Statistical tests were two-sided tests, and P-value <0.05 was considered statistically significant. All analyses used R language.

## Results

### Baseline Patients and Cycle Characteristics

**Figure 1:**
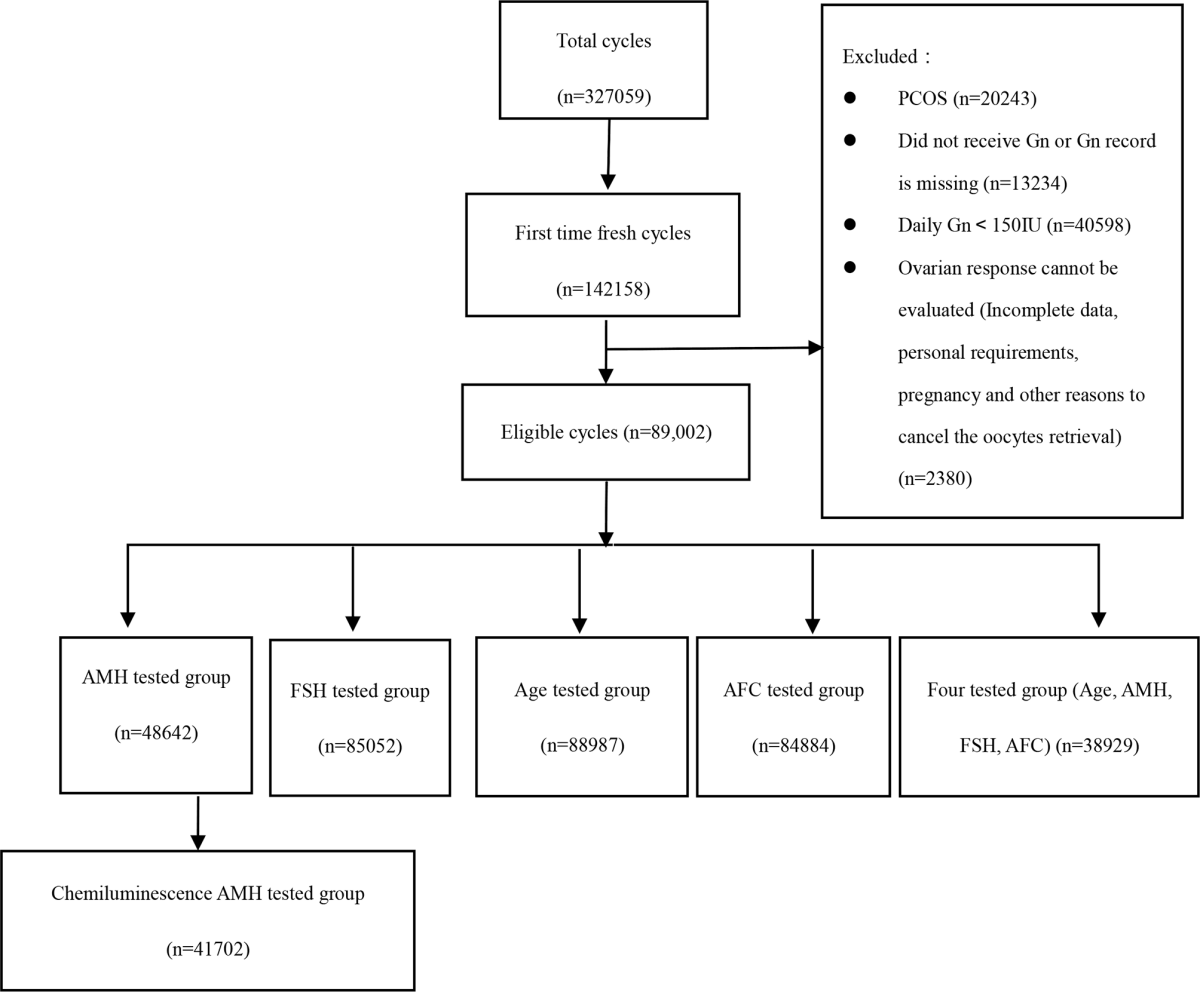
The data processing.

Five large- and medium-sized reproductive centers located in different regions of China (east, west, south, north, and middle area) conducted a total of 327,059 IVF/ICSI cycles, of which 145,158 (44.38%) were fresh cycles of first ovulation induction, and at last 89,002 cycles were eligible. Among them, 48,642 cases (54.65%) had AMH test results, 41,702 cases (46.86%) of which used the same detection method (electrochemiluminescence method, Kangrun Biotech); 85,052 cases (95.56%) had bFSH test results; 88,987 cases (99.98%) were recorded with age; 84,884 cases (58.47%) were recorded with AFC. The specific inclusion and exclusion process of patients is shown in **Figure 1**.

**Table 1** describes the baseline characteristics of these patients, including demographic information and ORT parameters. All study subjects were Chinese women (N=89,001), with an average age of 32.0 ± 5.1 years and an average BMI of 22.4 ± 3.1. Women over 35 years old accounted for 23.9%, and women over 38 years old accounted for 12.1% of the study population. The most common indications of IVF treatment were pelvic tubal factors (47.1%), male factors (15%), and ovarian factors (14.6%). The average AMH level was 3.6 ± 3.0 ng/ml, the average AFC was 11.1 ± 5.5, and the average bFSH was 7.7 ± 3.3 mIU/ml. Commonly used ovulation induction protocols included GnRH agonist protocol (69.1%), GnRH antagonist protocol (22.2%), progestin-primed protocol (2.2%), and COS protocol without ovarian suppression (6.55%). The median total dose of Gn was 2,200.0 [1725.0,2775.0] IU (quartile), the average was 2,320.4 ± 922.0 IU; the median Gn use days was 10.0 [9.0,11.0] days (quartile), and the average is 10.1 ± 2.5 days. During the process of Gn usage, recombined FSH accounted for 54.01%, and HMG was added to the latter stage of COS in 97.6% of the cycles. The average daily Gn average was 228.5 ± 65.8 IU. POR occurred in 13,196 patients (14.8%).

**Table 1:** Baseline characteristics and treatment outcome of the 89,001 women in the study group.

Participant characteristics	Mean ± SD
Female age*	32.0 ± 5.1
Female BMI*	22.4 ± 3.1
Infertility duration*	4.0 ± 10.3
Infertility factors*, No. (%)	
** Ovary factor**	11,963 (14.6)
** Male factor**	12,268 (15.0)
** Pelvic or tubal factor**	38,586 (47.1)
** Genetic factor**	3,209 (3.9)
** Uterine or cervix factor**	6,283 (7.7)
** Endometriosis**	6,014(7.3)
** Other factors**	3,570 (4.4)
Basal AMH*, Mean ± SD	3.6 ± 3.0
BasalE2*, Mean ± SD	47.6 ± 29.6
AFC*, Mean ± SD	11.1 ± 5.5
Basal bFSH*, Mean ± SD	7.7 ± 3.3
COS Protocols*, No. (%)	
** GnRH agonist protocol**	57,630 (69.1)
** GnRH antagonist protocol**	18,512 (22.2)
** Progestin-primed protocol**	1,875 (2.2)
** No ovary suppression**	5,437 (6.55)
**POR, No. (%)**	
** No**	75,805 (85.2)
** Yes**	13,196 (14.8)

*Data not available for all subjects. Missing values: female age = 14, female BMI = 647, infertility duration = 4,179, female age group = 14, female. BMI group = 647, infertility type= 1,711, infertility factor = 7,108, protocol group = 5,547, AFC = 4,117.

### Regression Analysis and ROC Curve

Multivariate logistic regression showed that age was significantly associated with POR with an odds ratio (OR) (95% CI) of 1.050 (1.040–1.059); AFC, AMH, and bFSH were also significantly associated with POR with OR (95% CI) of 0.898 (0.886–0.912), 0.712 (0.672–0.754), and 1.090 (1.073–1.106), respectively (P<0.001) (shown in affiliated table). Age, an independent influencing factor on pregnancy outcomes, was correlated with the other predictors in the study, so we created two models for the prediction of POR. The first models were univariate models for each of the ORTs (bFSH, AFC, or AMH) and age as predictors separately. The second models were multivariate models that evaluated combinations of each ORT and age together. The different models were used to parse out the predictive value of age and ORTs alone, as well as the added predictive value of age to AMH, AFC, bFSH in combination. ORT parameters combined with age significantly improved the prediction of POR after control ovarian stimulation (COS) (see in **Table 2**).

**Table 2:** Univariate and multivariate models of age and ORT in the prediction of POR.

	N	OR (95% CI)	p-value
**Univariate models**			
Age (per year)	88,987	1.183 (1.179–1.188)	<.0001
bFSH (per IU/L)	85,052	1.258 (1.250–1.266)	<.0001
AFC (per N)	84,884	0.707 (0.702–0.711)	<.0001
AMH (per ng/ml)	41,702	0.370 (0.359–0.382)	<.0001
**Multivariate models**			
**Age and bFSH**			
Age (per year)	85,041	1.164 (1.159–1.169)	<.0001
bFSH (per IU/L)	85,041	1.219 (1.211–1.227)	<.0001
**Age and AFC**			
AFC (per N)	84,872	0.736 (0.731–0.741)	<.0001
Age (per year)	84,872	1.086 (1.081–1.091)	<.0001
**Age and AMH**			
Age (per year)	41,695	1.084 (1.077–1.090)	<.0001
AMH (per ng/ml)	41,695	0.412 (0.400–0.425)	<.0001

Based on the results from the models in **Table 2**, we constructed the ROC curve for each factor of the ORTs and the combination of age and each ORTs that predicted POR with statistical significance. Next, we compared the area under the curve. Due to the nature of this retrospective analysis, not all parameters of each patient were complete. Therefore, we drew the ROC curve on (i) the whole population (whole group) (N=89,001) and (ii) the patients with complete data (four tested groups) (N=38,929). As the detection of AMH was updated from the previous ELISA method to the current electrochemiluminescence method, we only studied patients whose AMHs were measured by electrochemiluminescence (41,702 cases) to exclude the influence of different detection methods.

The multivariate analysis of POR prediction showed that the prediction accuracy in the combined model with all predictors AFC, AMH, bFSH, and age was higher than that of the models based on only one parameter. The AUC (95% CI) of the combined model was 0.873 (0.868–0.879). The AUC of the combined model was significantly better than the predicted value of a single parameter, but not significantly better than AMH plus age with AUC (95% CI) of 0.865 (0.860–0.870). The model with AMH alone had the highest AUC (AUC 0.862) among the univariate prediction models; followed by AFC (AUC 0.842). Adding age to the AMH model did not significantly improve the prediction accuracy (AUC 0.865 with age *vs.* AUC 0.862 without age). On the other hand, the age plus AFC model significantly improved the prediction accuracy of the single AFC model (AUC 0.846 with age *vs.* AUC 0.837 without age). The AUC of bFSH was relatively small in comparison to the other predictors, AUC 0.689 (0.683–0.695), and the predictive effects of single and combined bFSH use were both moderate. Details can be seen in **Table 3**.

**Table 3:** AUCs of prediction models of age and ORTs for the prediction of POR.

	Total group		Four-tested group	
ROC Model	AUC (95% CI)	n	AUC (95% CI)	n
**Univariate models**				
Age	0.723 (0.718–0.728)	88,987	0.712 (0.704–0.720)	38,929
bFSH	0.689 (0.683–0.695)	85,052	0.681 (0.673–0.690)	38,929
AFC	0.842 (0.838–0.846)	84,884	0.837 (0.832–0.843)	38,929
AMH	0.862 (0.857–0.867)	41,702	0.858 (0.852–0.864)	38,929
**Multivariable models**				
Age+bFSH	0.773 (0.769–0.778)	85,041	0.765 (0.757–0.772)	38,929
Age+AFC	0.850 (0.846–0.854)	84,872	0.845 (0.839–0.850)	38,929
Age+AMH	0.865 (0.860–0.870)	41,695	0.862 (0.856–0.867)	38,929
Age+bFSH+AFC+AMH	0.873 (0.868–0.879)	38,929	0.873 (0.868–0.879)	38,929

**Figure 2:**
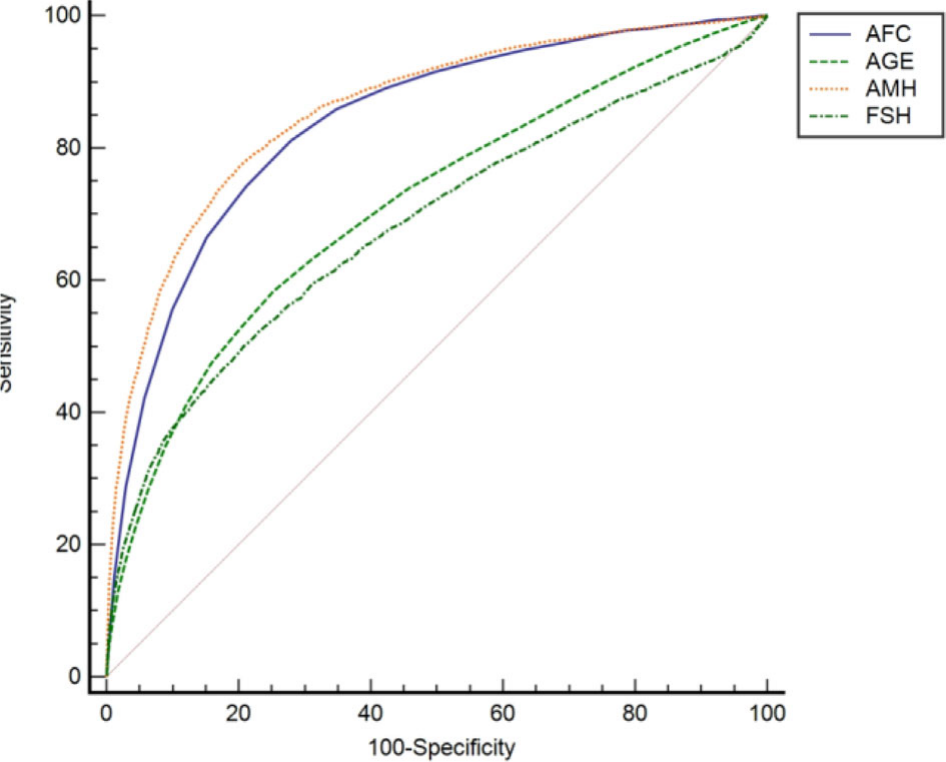
ROC curves of the POR prediction model by each parameter.

ROC curves of univariate and multivariate models are shown in **Figure 2**.

### Cutoff Points

For each predictor, the cutoff point was determined based on specificity of about 90% for predicting POR. We report the values for the predictors at 90% specificity and their sensitivities. The ROC analysis found that to predict POR, a cutoff point of 38 years old yielded a sensitivity of 40.7%; the cutoff point of bFSH was 9.8 mIU/ml with a sensitivity of 38.4%; the cutoff point of AFC was 5 with a sensitivity of 55.9%; the cutoff point of AMH was 1.18 ng/ml with a sensitivity of 63.3%. Comparing these factors used independently for POR prediction, AMH achieved the highest sensitivity with 90% specificity. AMH levels below 1.18 ng/ml were associated with a higher incidence of POR. After stratifying by age group, for patients younger than 35 years old, the cutoff point for AMH is 1.37 ng/ml and for AFC is 6. Details can be seen in **Table 4**.

**Table 4:** Cutoff point analysis—total group and age stratification.

Variable	Cutoff point	Sensitivity	Specificity	Youden index
**Age**				
**Total**	≤38	0.407	0.890	0.296
**bFSH**				
**Total**	≤9.8	0.384	0.900	0.283
**<35**	≤9.62	0.354	0.900	0.254
**35–38**	≤10.18	0.351	0.900	0.251
**38–40**	≤10.49	0.362	0.900	0.262
**>40**	≤11.51	0.320	0.900	0.220
**AFC**				
**Total**	≤5	0.559	0.908	0.467
**<35**	≤6	0.538	0.895	0.434
**35–38**	≤4	0.377	0.925	0.303
**38–40**	≤3	0.319	0.933	0.252
**>40**	≤3	0.465	0.875	0.340
**AMH**				
**Total**	≤1.18	0.633	0.900	0.534
**<35**	≤1.37	0.607	0.900	0.508
**35–38**	≤1.02	0.538	0.901	0.438
**38–40**	≤0.8	0.493	0.899	0.392
**>40**	≤0.61	0.518	0.899	0.417

## Discussion

These results of this real-world study, based on a multicenter retrospective study of 89,002 patients, indicate that age, AFC, AMH, and bFSH are predicting factors for POR, of which AMH and AFC are the best indicators when using a single factor for prediction. Age improves the above predictions with a cutoff point of 38 years old. When testing is reliable, AMH can be used alone to forecast POR. However, while AFC is used as a prediction parameter, we suggest that female age should be included at the same time for reference.

In the long-lasting debate on the true value of ORTs prior to IVF, a real-world study can be of help as an objective approach in summarizing the available evidence. The real-world study results are more representative of usual clinical practice and have important guiding significance for clinical practice (19). With the cooperation of various centers, we were able to collect a great number of cases with good homogeneity and were able to explore prediction of POR in Chinese infertile populations. In addition, this study adds to a body of literature describing predictors of POR that have historically been defined according to the Bologna criteria and the Poseidon criteria. Furthermore, we screened instruments, methods, and reagents of AMH measurement to accommodate the heterogeneity between centers.

The findings from our analysis confirm those of previous systematic reviews and meta-analysis of both single ORTs and multivariable prediction models for POR to control ovarian stimulation (6, 20, 21). Both AMH and AFC strongly represent the size of the cohort of FSH-sensitive follicles in the ovaries, thus often referred to as the quantitative ovarian reserve. AFC and AMH are highly correlated and also have discordance (22, 23). Comparing AMH with AFC, AMH has the advantage of very little intra- and inter-cycle variability (24). When challenged against AFC, AMH level is not only a quantitative but also a qualitative follicle marker, in relation with clinical and endocrine parameters (25, 26). Through our study, we conclude that AMH is the best independent predictor of POR, when comparing other ORTs and age separately as predictors. Historically, there were issues with AMH’s low comparability of measured values between clinical laboratories; however, recent advances in new automated assays have greatly improved repeatability and comparability (27). For all the cases included in our model, AMH is tested by Access AMH with electrochemiluminescence detection, which is more accurate than the ELISA method (28, 29). The predictive effect of AFC has often been questioned because of variability in the operator’s technical proficiency. However, our study shows that across various centers, AFC was a good predictor of POR. This may be related to the fact that all the centers participating in the study are large reproductive centers in China, with well-trained sonographers, advanced ultrasound equipment, and standardized management. Nevertheless, age improves the prediction of AFC significantly.

The clinical use of markers like AMH, bFSH, and AFC is mostly based on cutoff points. From the individual patient dataset, cutoff points for poor response prediction could be derived that have general applicability. Unfortunately, cutoff points reported in literature are very variable (3, 11, 18). Such variability could be explained by factors such as the low number of subjects included in some of these studies, the variability in the measuring methods used for these markers, and the different definitions of POR. According to published studies, cutoff points of AMH range 0.10–1.66 ng/ml, with reported sensitivities of 44–97% and specificities of 41–100%; cutoff points of AFC range between 3–10, with reported sensitivities of 9–73%; and specificities of 73–100% (13, 17). Our study shows that for predicting POR, the cutoff points of AMH and AFC were 1.18 ng/ml and 5, respectively, for predicting POR in the whole population, ranging between 0.61–1.37 and 3–6 in different age groups, and decreased with age. For younger women (less than 35 years old and 35~38 years old), cutoff points of AMH and AFC were 1.37 and 1.02 ng/ml, 6 and 4. These results may help recognize and intervene in young patients with ovarian reserve decline. Recent publications have also suggested the calculation of age-specific ovarian reserve decline curves in order to maximize ORT accuracy (30, 31).

The cutoff point of age is 38 years (specificity 89%, sensitivity 40.7%, AUC 0.723), which differs from the existing 40 years old cutoff point in the Bologna criteria and 35 years old cutoff point in the Poseidon categories. It was also reported with given evidence from multiple studies that the average rate of follicular depletion, aneuploidy rate, and embryo arrest rate all increase significantly after age 38 (32–34).

A limitation of this study is that although Access AMH with electrochemiluminescence detection was used, variability between different laboratories in each center is still worth exploring. Also, pregnancy outcomes and effective management strategies of POR patients are not referred to. These should be explored on the basis of this research in the future.

In conclusion, POR is estimated to occur in 14.8% of the first IVF cycles in the Chinese population. When testing is reliable, AMH can be used alone to forecast POR. When AFC is used as a prediction parameter, age is suggested to be considered as well. AFC ≤5, AMH ≤1.18 ng/ml, and female age ≥38 should be recommended to predict POR more accurately in IVF/ICSI patients.

## Data Availability Statement

The raw data supporting the conclusions of this article will be made available by the authors, without undue reservation.

## Ethics Statement

The studies involving human participants were reviewed and approved by the Ethics Committee of Henan Provincial People’s Hospital, Ethics Committee of Tongji Hospital of Tongji Medical College of Huazhong University of Science and Technology, Ethics Committee of Jiangsu Provincial Hospital, Ethics Committee of Northwest Women’s and Children’s Hospital, and Ethics Committee of Sixth Affiliated Hospital of Sun Yat-sen University. Written informed consent for participation was not required for this study in accordance with the national legislation and the institutional requirements.

## Author Contributions

C-LZ and X-YL supervised the entire study, including the procedures, conception, design, and completion, and participated in the interpretation of the study data and in revisions to the article. LJ, J-ZS, Y-DM, and RH were responsible for the collection of data. XW contributed to the data analysis and drafted the article. Y-NJ wrote sections of the manuscript. All authors contributed to the article and approved the submitted version.

## Funding

The study was funded by National Natural Science Foundation of China (No. U2004130 http://www.nsfc.gov.cn/), and National Key R&D Program of China (2018YFC1002106).

## Conflict of Interest

The authors declare that the research was conducted in the absence of any commercial or financial relationships that could be construed as a potential conflict of interest.

## Publisher’s Note

All claims expressed in this article are solely those of the authors and do not necessarily represent those of their affiliated organizations, or those of the publisher, the editors and the reviewers. Any product that may be evaluated in this article, or claim that may be made by its manufacturer, is not guaranteed or endorsed by the publisher.

## Supplementary Material

Supplementary Table 1 Univariable and multivariable models of age and ORTs in the prediction of poor response.

Supplementary Table 2 Univariable and multivariable models of age and ORT in the prediction of poor response.
