# Supplementary material for: Evaluation of Ovarian Reserve Tests and Age in the Prediction of Poor Ovarian Response to Controlled Ovarian Stimulation—A Real-World Data Analysis of 89,002 Patients
Source: Front Endocrinol (Lausanne). 2021 Aug 30;12:702061. doi: 10.3389/fendo.2021.702061 (PMC8435745; doi:10.3389/fendo.2021.702061)
Supplement: Supplementary Table 1 — Univariable and multivariable models of age and ORTs in the prediction of poor response. [file Table_1.docx]

Supplementary table

Table1 Univariable and multivariable models of age and ORTs in the prediction of poor response

| Factors | N | Odds Ratio  (Univariate Model) | P-Value(Univariate Model) | Addjusted Odds Ratio (Multivariate Model) | Addjusted P-Value(Multivariate Model) |
| --- | --- | --- | --- | --- | --- |
| Infertility type |  |  |  |  |  |
| Secondary infertility | 43035 | ref |  | ref |  |
| Primary infertility | 44255 | 0.593(0.571-0.616) | <0.001 | 1.177(1.073-1.292) | <0.001 |
| Total | 87290 |  |  |  |  |
| **COS Protocols*, No. (%)** |  |  |  |  |  |
| GnRH agonist protocol | 57629 | ref |  | ref |  |
| GnRH antagonist protocol | 18513 | 3.571(3.398-3.753) | <0.001 | 1.184(1.061-1.321) | 0.0025 |
| Progestin-primed protocol | 1876 | 17.536(15.917-19.319) | <0.001 | 1.972(1,657-2.347) | <0.001 |
| Mild stimulation | 5308 | 23.358(21.896-24.918) | <0.001 | 3.158(2.724-3.661) | <0.001 |
| No ovary suppression | 129 | 40.250(27.269-59.413) | <0.001 | 4.379(1.906-10.061) | <0.001 |
| Total | 25826 |  |  |  |  |
| **Infertility factors*, No. (%)** |  |  |  |  |  |
| male factor | 12268 | ref |  | ref |  |
| ovary factor | 11963 | 15.992(14.753-17.335) | <0.001 | 2.050(1.701-2.471) | <0.001 |
| pelvic or tubal factor | 38586 | 1.389(1.282-1.506) | <0.001 | 1.141(0.951-1.368) | 0.1556 |
| genetic factor | 3209 | 1.265(1.092-1.465) | 0.0017 | 1.056(0.816-1.365) | 0.6798 |
| uterine or cervix factor | 6283 | 1.897(1.706-2.109) | <0.001 | 1.039(0.841-1.283) | 0.7228 |
| endometriosis | 6014 | 1.950(1.753-2.169) | <0.001 | 1.745(1.393-2.187) | <0.001 |
| other factors | 3570 | 1.46(0.991-1.325) | 0.0664 | 1.353(1.020-1.794) | 0.0358 |
| Total | 81893 |  |  |  |  |
