# Supplementary material for: Evaluation of Ovarian Reserve Tests and Age in the Prediction of Poor Ovarian Response to Controlled Ovarian Stimulation—A Real-World Data Analysis of 89,002 Patients
Source: Front Endocrinol (Lausanne). 2021 Aug 30;12:702061. doi: 10.3389/fendo.2021.702061 (PMC8435745; doi:10.3389/fendo.2021.702061)
Supplement: Supplementary Table 2 — Univariable and multivariable models of age and ORT in the prediction of poor response. [file Table_2.docx]

Supplementary table

Table2 Univariable and multivariable models of age and ORT in the prediction of poor response

| Factors | N | Odds Ratio  (Univariate Model) | 1. Value   (Univariate Model) | Addjusted Odds Ratio (Multivariate Model) | Addjusted P-Value(Multivariate Model) |
| --- | --- | --- | --- | --- | --- |
| Female age | 88988 | 1.183(1.179-1.188) | <0.001 | 1.050(1.040-1.059) | <0.001 |
| Female BMI | 88355 | 1.044(1.038-1.050) | <0.001 | 1.050(1.035-1.065) | <0.001 |
| Basal AMH | 41702 | 0.370(0.351-0.391) | <0.001 | 0.712(0.672-0.754) | <0.001 |
| Basal bFSH | 85052 | 1.258(1.247-1.269) | <0.001 | 1.090(1.073-1.106) | <0.001 |
| Basal LH | 84995 | 1.007(1.002-1.012) | 0.0080 | 1.001(0.988-1.014) | 0.8799 |
| AFC | 84884 | 0.707(0.701-0.712) | <0.001 | 0.898(0.886-0.912) | <0.001 |
| Total Gn does | 89002 | 1.000(1.000-1.000) | <0.001 | 1.000(1.000-1.000) | <0.001 |
| Total Gn day | 89002 | 0.707(0.699-0.715) | <0.001 | 0.778(0.750-0.807) | <0.001 |
